# Melanocytes in the Skin – Comparative Whole Transcriptome Analysis of Main Skin Cell Types

**DOI:** 10.1371/journal.pone.0115717

**Published:** 2014-12-29

**Authors:** Paula Reemann, Ene Reimann, Sten Ilmjärv, Orm Porosaar, Helgi Silm, Viljar Jaks, Eero Vasar, Külli Kingo, Sulev Kõks

**Affiliations:** 1 Department of Physiology, Institute of Biomedicine and Translational Medicine, University of Tartu, Tartu, Estonia; 2 Core Facility of Clinical Genomics, Department of Pathophysiology, Department of Biomedicine, Institute of Biomedicine and Translational Medicine, University of Tartu, Tartu, Estonia; 3 The Institute of Veterinary Medicine and Animal Sciences of the Estonian University of Life Sciences, Competence Centre on Reproductive Medicine and Biology, Tartu, Estonia; 4 Department of Physiology, Institute of Biomedicine and Translational Medicine, University of Tartu and Quretec Ltd (private limited company), Tartu, Estonia; 5 Department of Pediatric Surgery, Tallinn Children's Hospital, Tallinn, Estonia; 6 Clinic of Dermatology, Tartu University Hospital, Department of Dermatology, University of Tartu, Tartu, Estonia; 7 Institute of Molecular and Cell Biology, University of Tartu, Tartu, Estonia; 8 Department of Physiology, Institute of Biomedicine and Translational Medicine, University of Tartu and Centre of Translational Medicine, University of Tartu, Tartu, Estonia; 9 Department of Pathophysiology, Institute of Biomedicine and Translational Medicine, University of Tartu and Centre of Translational Medicine, University of Tartu, Tartu, Estonia; IDI, Istituto Dermopatico dell'Immacolata, Italy

## Abstract

Melanocytes possess several functions besides a role in pigment synthesis, but detailed characteristics of the cells are still unclear. We used whole transcriptome sequencing (RNA-Seq) to assess differential gene expression of cultivated normal human melanocytes with respect to keratinocytes, fibroblasts and whole skin. The present results reveal cultivated melanocytes as highly proliferative cells with possible stem cell-like properties. The enhanced readiness to regenerate makes melanocytes the most vulnerable cells in the skin and explains their high risk of developing into malignant melanoma.

## Introduction

Skin is a highly organized and differentiated structure, which consist of various cell types. Keratinocytes (KC) and fibroblasts (FB) together form the majority of cellular components in the skin (7×10^5^–9×10^5^ KC per mm^2^ [1] and 4×10^3^ mid-dermis FB to 10^5^ papillary FB per mm^3^ [2], [3]). Therefore the functional properties of the highly outnumbered melanocytes (MC) have received relatively less attention. The average number of pigment-producing MC depends on the body site and is estimated to be between 500 to 2000 MC per mm^2^ [1], [4]. Interestingly, differences in ethnic background manifest in the intensity of melanogenesis and the morphology of dendrites, but not in the quantity of MC [5]. Despite their small number, MC have proven to have several roles besides melanogenesis, a well-characterized property of MC. They are able to secrete a wide range of signaling molecules, e.g. proinflammatory cytokines, immunosuppressive molecules, neuromediators etc. [2], [6]–[9]. MC interacts highly with surrounding KC, which have been shown to regulate MC survival, dendricity, melanogenesis, and the expression of cell surface receptors [10].

Numerous gene expression analyses of different skin cell populations have been performed in both physiological and pathological states using an array of detection techniques ranging from quantitative real time polymerase chain reaction (qPCR) and in situ hybridization to high throughput methods such as serial analysis of gene expression and microarrays [11]–[13]. However, all these methods have specific limitations. In contrast, the use of high-throughput RNA-Seq on rRNA-depleted samples allows the detection of nearly all coding and non-coding RNA species in a given sample.

In the present study we outline, to our knowledge for the first time, the differences of MC compared to other main cell types of the skin at the level of complete transcriptome. We used whole skin samples and cultivated primary skin cells, harvested from the same body site of healthy subjects of similar age.

## Materials and Methods

### Ethics Statement and Patients

All procedures were carried out in accordance with the ethical standards. This study (including written consent form) has been approved by Research Ethics Committee of the University of Tartu (approval number 178/T-19). The patients with no concurrent diseases and signs of infection, were recruited from among elective patients present at the Department of Pediatric Surgery, Tallinn Children's Hospital. A written informed consent was obtained from all parents or caretakers of patients under 18 years. Additionally, separate written informed consent was obtained from all patients aged 8–17 years. Nine pediatric foreskins from healthy volunteers (aged 5 months to 10 year) were used. Collected tissue samples were by-products of circumcise procedures and no additional intervention was caused by our investigation. All samples were coded and information of the donor identity was only available for the physician. All patient related information was stored separately from the samples and the data.

### Cell culture

From each tissue sample three skin cell types (keratinocytes, melanocytes and fibroblasts) were harvested. After rinsing in phosphate-buffered saline (PBS w/o Ca, mg, PAA Laboratories), subcutaneous fat was removed from skin pieces and tissues were incubated in dispase II (2.4 U/ml, Sigma-Aldrich) at +4°C overnight. Epidermis was peeled off from the dermis, transferred into 0.05% trypsin/0.02% EDTA (Life Technologies) for 3 min at 37°C. Enzymatic digestion was stopped by adding the trypsin inhibitor (Sigma-Aldrich). MC culture dishes were precoated with gelatin before cell seeding. EpiLife basal medium with human keratinocyte growth supplement (Life Technologies) and melanocyte growth medium M2 with supplement mix (PromoCell) were used to cultivate corresponding cells.

A piece of dermis was used for isolation of FB by migration method as follows. The dermis was cut into 4×4 mm pieces and attached onto a culture dish, covered with 10 ml Dulbecco's Modified of Eagle's Medium (DMEM) (PAA Laboratories) supplemented with10% foetal bovine serum (Sigma-Aldrich). The medium was changed every 2nd day throughout the study. The skin cells were cultivated at low passage number (2–3) to minimize the influence of culturing conditions.

### RNA Extraction and Library Preparation and Whole Transcriptome Sequencing

Cultivated skin cells underwent to lysis and RNA extraction process using Trizol® (Invitrogen) as described in [12]. The skin biopsies were homogenized using Precellys® 24 system and previously optimized protocol [12] were used for total RNA extraction, followed by DNAse I (Qiagen) treatment. The purity and concentration of samples was checked with both Qubit spectrophotometer and Nano Drop ND-1000 and the integrity of the RNA (RIN) was evaluated using Agilent 2100 Bioanalyzer. We chose 12 total RNA samples, with the highest RIN (9–10), extracted from 4 KC, 4 MC, 2 FB and 2 whole skin samples for library preparation. Extracted mRNA was enriched using RiboMinusTM Eukaryote kit (Invitrogen) according to manufacturer's instructions. The final quantity of RNA was 10 µg per reaction. The cDNA library with size-selected in the range of 150–250 bp and following bar-coding preparation procedure was based on a protocol provided by Applied Biosystems. Samples were sequenced using SOLiD 5500xl platform with 75 bp forward and 35 reverse primers.

### Analysis of RNA-Seq data

Sequencing of cDNA libraries resulted in 24842284 to 44324428 paired reads per sample. For greater mapping quality the initial 75 bp F3 and 35 bp F5 reads were trimmed to 45 and 25 base pairs, respectively. All color-spaced reads were aligned to human reference genome (Ensembl, release 73) using TopHat v2.1.0 [14] that used Bowtie version 1.0.0 [15]. RPKM (reads per kilobase of transcript per million mapped reads) values for gene expression levels were calculated with Cufflinks v2.0.2 [16] and raw counts were retrieved with HTSeq version 0.5.3p9 (http://www-huber.embl.de/users/anders/HTSeq/) using gene annotations of protein coding genes downloaded from Ensembl (release 73). Differential expression was estimated on raw counts with edgeR [17]. All programs were used with their default parameters with TopHat set to not to find novel junctions.

### Modeling background regions

To estimate the number of truly expressed genes we modeled intergenic regions using a methodology described in [18]. Models of intergenic regions are expected to reflect the level of background expression (noise), which is taken as the baseline when estimating the number of expressed genes. For each gene, the length of the background region was equal to the gene's longest combined transcript (the sum of all transcribed nucleotides) and it extended upstream from position-1000 relative to the transcription start site. Only background regions that did not overlap with any expressed sequence tags (EST) were used in the analysis. A gene was considered as expressed only if the RPKM value in all samples of the corresponding cell type was above the cutoff (0.95). Conversely, the gene was labeled as not expressed if the RPKM value was below 0.95 in at least one of the samples. EST annotations were downloaded using UCSC Table Browser (http://genome.ucsc.edu/cgi-bin/hgTables).

### Differential expression analysis of gene expression

Differential expression was estimated between MC and samples from KC, FB and the whole skin using edgeR [17]. A gene was considered as differentially expressed if the FDR-adjusted p-value was below 0.05 and if the gene was expressed in at least one of the cell types.

To identify a gene as expressed only in MC and not in KC, FB (termed as “uniquely expressed in MC”) it had to meet the following requirements: 1) RPKM >0.95 in MC (gene is labeled as expressed in MC), 2) RPKM <0.95 in KC, FB (gene is labeled as not expressed in KC, FB), 3) differential expression FDR <0.05 (gene is differentially expressed in MC with respect to KC and FB).

### Pathway analysis of differentially expressed genes

Gene ontology enrichment analysis of differentially expressed genes was performed using g:GOSt (http://biit.cs.ut.ee/gprofiler/index.cgi) [19]. Bases on the p-values of g:GOSt analysis, GOsummaries package were used to generate wordclouds of gene names (http://cran.r-project.org/web/packages/GOsummaries/index.html). The word sizes in wordclouds are defined by the p-values.

Additionally, multidimensional scaling test for visualizing the level of similarity of individual samples in study groups were performed using edgeR. The results confirm the homogenity and purity of cell populations (S1 Fig.).

## Results and Discussion

### Overall differences between cultivated MC, KC, FB and whole skin tissue

As expected, the total number of expressed genes was the highest in whole skin samples (10,871 genes), since other cell types besides KC, MC and FB (epithelial cells, Merkel cells, Langerhans cells etc.) are found in a skin biopsy (Table 1). Interestingly, the total number of expressed genes was the lowest in KC (Table 1). Thereat, 7766 genes were commonly expressed in all study groups (whole skin and KC, MC and FB). The list of the detected genes and their RPKM values can be found in the S1 Table. Similarly, the number of genes considered as uniquely expressed was the largest in the whole skin sample (290 genes, Table 1). When comparing specific cell populations, FB had a higher number of uniquely expressed genes compared to MC and KC (277, 122, 138 uniquely expressed genes, respectively) (Table 1). It is likely that the true number of uniquely expressed genes is higher as we applied a relatively strict cutoff criterion (RPKM >0.95) when calling gene expression as present or absent. The genes, uniquely expressed in MC are displayed in Table 2.

**Table 1:** The number of detected and uniquely expressed genes.

Skin	Keratinocytes	Melanocytes	Fibroblasts
**Total number of expressed genes**			
10871	8937	9903	10420
**Uniquely expressed genes**			
290	138	122	277

**Table 2:** Uniquely expressed genes in MC.

Uniquely expressed genes in melanocytes							
Symbol	Gene Name	Symbol	Gene Name	Symbol	Gene Name	Symbol	Gene Name
ADCK1	aarF domain containing kinase 1	EME1	essential meiotic endonuclease 1 homolog 1 (S. pombe)	LSM11	LSM11, U7 small nuclear RNA associated	SCG2	secretogranin II
ADCY2	adenylate cyclase 2 (brain)	ENTHD1	ENTH domain containing 1	LYPD1	LY6/PLAUR domain containing 1	SEPT4	septin 4
ANKRD37	ankyrin repeat domain 37	EOMES	eomesodermin	LZTS1	leucine zipper, putative tumor suppressor 1	SFMBT2	Scm-like with four mbt domains 2
ANO5	anoctamin 5	EPHA5	EPH receptor A5	MC1R	melanocortin 1 receptor	SHC4	SHC family, member 4
ARL9	ADP-ribosylation factor-like 9	ESR2	estrogen receptor 2 (ER beta)	MCF2	MCF.2 cell line derived transforming sequence	SHROOM4	shroom family member 4
ASB9	ankyrin repeat and SOCS box containing 9	EVI2A	ecotropic viral integration site 2A	MCOLN2	mucolipin 2	SLAMF9	SLAM family member 9
BAIAP2L2	BAI1-associated protein 2-like 2	EVI2B	ecotropic viral integration site 2B	MDGA2	MAM-containing glycosylphosphatidylinositol anchor 2	SLC16A10	solute carrier family 16, member 10
BCL2	B-cell CLL/lymphoma 2	FABP7	fatty acid binding protein 7, brain	MGAT5B	mannosyl-glucosaminyltransferase, isozyme B	SLC19A1	solute carrier family 19 (folate transporter), member 1
BHLHE41	basic helix-loop-helix family, member e41	FAM124A	family with sequence similarity 124A	MMP8	matrix metallopeptidase 8 (neutrophil collagenase)	SLC19A3	solute carrier family 19, member 3
BMPR1B	bone morphogenetic protein receptor, type IB	FAM69B	family with sequence similarity 69, member B	NPM2	nucleophosmin/nucleoplasmin 2	SLC22A18AS	solute carrier family 22, member 18 antisense
BST2	bone marrow stromal cell antigen 2	FAXC	failed axon connections homolog (Drosophila)	NR4A3	nuclear receptor subfamily 4, group A, member 3	SLITRK2	SLIT and NTRK-like family, member 2
C11ORF96	chromosome 11 open reading frame 96	FCGR2A	Fc fragment of IgG, low affinity IIa, receptor (CD32)	PAEP	progestagen-associated endometrial protein	SORBS1	sorbin and SH3 domain containing 1
C2ORF88	chromosome 2 open reading frame 88	FOXD3	forkhead box D3	PDE3A	phosphodiesterase 3A, cGMP-inhibited	SSUH2	ssu-2 homolog (C. elegans)
C8ORF46	chromosome 8 open reading frame 46	FOXRED2	FAD-dependent oxidoreductase domain containing 2	PDE7B	phosphodiesterase 7B	ST6GALNAC3	N-acetylgalactosaminide alpha-2,6-sialyltransferase 3
CA8	carbonic anhydrase VIII	FRMD5	FERM domain containing 5	PDLIM3	PDZ and LIM domain 3	ST8SIA1	Alpha-N-Acetyl-Neuraminide Alpha-2,8-Sialyltransferase 1
CADM3	cell adhesion molecule 3	GAPDHS	glyceraldehyde-3-phosphate dehydrogenase, spermatogenic	PGBD5	piggyBac transposable element derived 5	TCN1	transcobalamin I (vitamin B12 binding protein
CD200	CD200 molecule	GJB1	gap junction protein, beta 1, 32kDa	PKN3	protein kinase N3	TFF3	trefoil factor 3 (intestinal)
CDH19	cadherin 19, type 2	GOLGA7B	golgin A7 family, member B	PKNOX2	PBX/knotted 1 homeobox 2	THEM6	thioesterase superfamily member 6
CGREF1	cell growth regulator with EF-hand domain 1	GPR19	G protein-coupled receptor 19	PLA1A	phospholipase A1 member A	TLR1	toll-like receptor 1
CHRNA6	cholinergic receptor, nicotinic, alpha 6 (neuronal)	GPRIN3	GPRIN family member 3	PLEKHH1	pleckstrin homology domain containing, family H, 1	TMEM169	transmembrane protein 169
CMPK2	cytidine monophosphate (UMP-CMP) kinase 2	GREB1	Growth regulation by estrogen in breast cancer 1	PPM1H	protein phosphatase, Mg2+/Mn2+ dependent, 1H	TMEM229B	transmembrane protein 229B
CRISPLD1	cysteine-rich secretory protein LCCL domain containing 1	HELZ2	helicase with zinc finger 2, transcriptional coactivator	PRDM7	PR domain containing 7	TMEM56	transmembrane protein 56
CSGALNACT1	chondroitin sulfate N-acetylgalactosaminyltransferase 1	HOXB7	homeobox B7	PRKCB	protein kinase C, beta	TMEM71	transmembrane protein 71
CSPG4	chondroitin sulfate proteoglycan 4	HPDL	4-hydroxyphenylpyruvate dioxygenase-like	RAB20	RAB20, member RAS oncogene family	TMPRSS5	transmembrane protease, serine 5
CTTNBP2	cortactin binding protein 2	HSF4	heat shock transcription factor 4	RNF157	ring finger protein 157	TRIM6	tripartite motif containing 6
CXORF57	chromosome X open reading frame 57	IL16	interleukin 16	RNF182	ring finger protein 182	TSPAN10	tetraspanin 10
CYTL1	cytokine-like 1	ITPR1	inositol 1,4,5-trisphosphate receptor, type 1	ROPN1	rhophilin associated tail protein 1	TTYH2	tweety homolog 2 (Drosophila)
DISC1	disrupted in schizophrenia 1	KCNN2	potassium intermediate/small conductance calcium-activated channel, subfamily N, member 2	RTKN2	rhotekin 2	WDR17	WD repeat domain 17
DNMT3A	DNA (cytosine-5-)-methyltransferase 3 alpha	KIAA1211	KIAA1211	RTP4	receptor (chemosensory) transporter protein 4	ZNF280B	zinc finger protein 280B
DPY19L2	dpy-19-like 2 (C. elegans)	LPL	lipoprotein lipase	RUNX3	runt-related transcription factor 3		
EGFL8	Epidermal growth factor-like protein 8; Lysosomal thioesterase PPT2	LRRC45	leucine rich repeat containing 45	RXRG	retinoid X receptor, gamma		

The list of genes, expressed in MC, but not in KC and FB.

**Figure 1:**
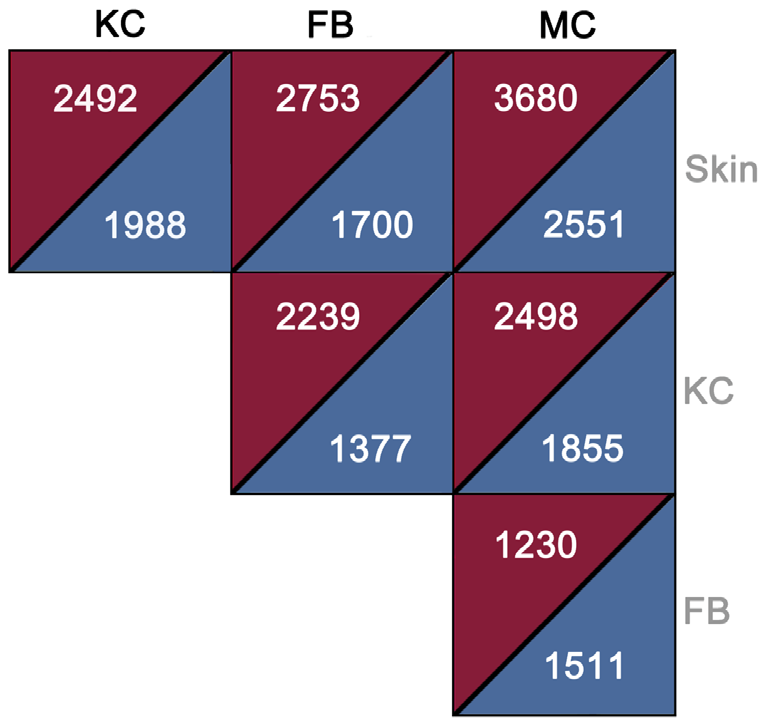
The number of differentially expressed genes in each study group - melanocyte (MC), keratinocyte (KC), fibroblast (FB) and whole skin (Skin). Red triangles – upregulated genes, blue triangles – downregulated genes.

Based on the differential gene expression analysis, we identified significantly fewer similarities between MC and whole skin gene expression patterns than when comparing KC or FB to the whole skin. In melanocytes, 6231 genes were differentially expressed compared to the whole skin. Of those, 3680 were upregulated in MC and 2551 downregulated in MC with respect to whole skin samples (Fig. 1). The number of differentially expressed genes with respect to whole skin was 4480 in KC and 4454 in FB. This finding can be explained by the relatively small proportion of MC in the total cell number of skin. The entire list of differentially expressed genes can be found in S2 Table.

### Differential gene expression in MC

Previous studies have mainly pointed out the role of melanocytes as pigment-producing cells in the skin. The gene expression profile of MC has been compared to other normal skin cells but also to pathologic melanoma cells [11], [13]. We confirmed the high expression level of previously identified melanocyte-specific genes such as DCT, TYR, KIT, EDNRB, MITF, and TYRP1, in MC compared to FB and KC (S2 Table), as reported also in a previous microarray study [11]. DCT, TYR and TYRP1 encode enzymes acting in the melanin synthesis pathway [20]. EDNRB and MITF are crucial for melanoblast proliferation and KIT is needed for the differentiation of melanoblasts into TYR-positive MC [21], [22]. We also showed MC1R, PLA1A, NPM2 to be uniquely expressed in MC, but not in KC or FB (Table 2) corroborating previously published data [23]. MC1R, a receptor for melanocyte-stimulating hormones and adrenocorticotropic hormone, is involved in regulating the pigmentation of the skin and hair. PLA1A and NPM2 have both been shown to be essential in melanoma progression [24], [25]. In agreement with previous studies [11], [13] the expression of ABCC2, DNAJA1, GPR143, MLANA, OCA2, QPCT, RRAGD, TBC1D7 and GPR137B was detected at a higher level in MC compared to KC and FB (S2 Table). Majority of these genes are also related to the melanogenesis pathway, controlling the growth and maturation of melanosomes, being involved in melanosome biogenesis, transporting melanin precursor molecules or being candidate genes in melanocytic tumor progression [26]–[30].

In the present study our goal was to detect other potential functions and key genes of MC. Comparing MC, KC and FB, we found several gene groups, which distinguish MC from other skin cells.

The most prominent group of genes differentially expressed in MC compared to FB (Fig. 1) was that of encoding various histone proteins (HIST1H1A, HIST1H1B, HIST1H2AA, HIST1H2AE, HIST1H2AG, HIST1H2AI, HIST1H2BB, HIST1H2BH, HIST1H2BI, HIST1H2BN, HIST1H3D, HIST1H3F, HIST1H3H, HIST1H3J, HIST1H4D, HIST1H4I, HIST1H4L, HIST2H3D, HIST3H2A, HIST3H2BB and HIST4H4) (S2 Table). Another group of genes, the expression of which drastically differed in MC compared to FB, were cell division cycle protein genes (CDC20, CDC25A, CDC25C, CDC6, CDCA2, CDCA5, CDCA8) (S2 Table). Additionally, a set of kinesin family genes (KIF13B, KIF20B, KIF21A, KIF22, KIF24, KIF2C, KIFC1 was differentially expressed in MC compared to FB (S2 Table). We saw also significantly higher expression of calcium-binding proteins S100A1, S100A14, S100A8, S100A9 and S100B genes in MC compared to FB (S2 Table).

Above mentioned gene groups, which are prominently expressed in MC but not in FB suggest that MC are active and intensively proliferative in the culture conditions. For instance, the high prevalence of histone genes in MC reveal to intensified DNA synthesis as histones are responsible for nucleosome structure and proper DNA wrapping [31]. This corresponds well with the high proliferative activity we saw during the cell cultivation process. This conclusion is substantiated by the increased expression of a number of cell cycle regulating genes, which are involved in the regulation of cell cycle at several steps and levels and kinesin genes, which are related to cell movements and intracellular trafficking, including chromosome and centrosome positioning during mitosis [32]. Calcium-binding proteins are responsible for numerous cellular processes, e.g. cell cycle regulation and differentiation, but have also been suggested to have tumor suppressor functions and are highly expressed in cells with stem cell properties [29], [33].

Compared to KC, MC expressed a set of major histocompatibility complex protein genes such as HLA-B, HLA-DMA, HLA-DPB1, HLA-DRA and HLA-F (S2 Table). In addition genes, which are related to viral and bacterial infection defense mechanism - interferon induced protein genes [34] IFI27, IFI35, IFI44, IFI44L, IFI6, IFIT1, IFIT2, IFIT3, IFITM1, IFITM2 and IFITM3, were highly expressed in MC compared to KC (S2 Table). KC have been shown to be the key players in modulating the immunological status of physiological and pathological skin; being the first sensors of harmful agents, they secrete inhibitory and stimulating cytokines, and activate other immune competent cells (e.g. Langerhans cells) [35]. Our data suggest that the role of MC in cutaneous immune system regulation might be more extensive than anticipated so far.

Importantly, we could identify a set of MC-specific genes previously not described by other researchers. In many cases the functions of these genes are poorly characterized and further experiments are needed to identify their precise role in MC (Table 2).

A number of genes that were unique for MC (CA8, CHRNA6, CTTNBP2, EPHA5, FAXC, KCNN2, SCG2, SLITRK2) (Table 2) refer to MC's origin from the neural crest [36]–[44]. Additionally, a number of genes (ANO5, CGREF1, EGFL8, ENTHD1 and ITPR1) involved in calcium-mediated processes were also uniquely expressed in MC (Table 2).

Next we identified a number of genes that were not uniquely expressed in MC but were specifically upregulated in melanocyte culture. Their specific role in melanocytes is unclear, but based on the existing biological data these genes can be divided into following functional classes: tumorogenesis, inflammation and stem cell related genes.

#### Melanocytes and tumorogenesis

Genes belonging to pathways involved in tumor progression are more characteristic to MC than to KC and FB. High expression of tumor suppressive genes [45]–[48] and its candidate genes such as ARL9, C10orf90, SLC22A18, DAPK1, BEX1, PYHIN1, IGSF8 could be observed in MC compared to KC and FB (S2 Table). Interestingly, a novel tumor suppressor IGLON family genes (IGLON2, IGLON 3 and IGLON4) were all detectable in MC. IGLON3 also known as LSAMP was prominently expressed in MC compared to KC and FB (S2 Table). IGLON family genes are mainly described as regulators of nerve growth factors but recent studies have shown their strong tumor suppressive capacities [49].

A number of genes, which normally have a role in growth and dividing processes or in apoptosis regulation, can play a role in cancer formation. For example several RING-type zinc finger proteins, which function to avoid uncontrolled proliferation and to be a part of embryonic development, act as cancer development modulators [50]. As an example, certain RING finger proteins RNF144A, RNF157 and RNF187 were specifically upregulated in MC (S2 Table). DNAJA1 is increased in MC (S2 Table), which overexpression reduces cancer cell survival [51].

Interestingly, laminin alpha 1 gene LAMA1 was highly expressed in MC compared to KC and FB (S2 Table). Laminins are integral parts of the extracellular matrix [52]. LAMA1 is present mostly in the early stages in most tissues of the embryo and is not common for adult tissues [53]. These results suggest that MC might have an important role in basement membrane formation and remodeling and might refer to a higher potential of MC to transform into tumorous cells.

The family of sialyltransferases, which comprises a large group of enzymes, responsible for the synthesis of sialylated glycans, regulates immune response including virus binding [54]. Sialylated glycans can be found on the surface of many tumor cells where they counteract the recognition of malignant cells by the immune system [55]. Our analysis identified several sialyltransferase genes ST3GAL4, ST3GAL5, ST3GAL6, ST6GAL1, ST6GALNAC3, ST8SIA1 and ST8SIA6, which were expressed at a higher level in MC compared to KC and FB (S2 Table).

Further, we confirmed a differential expression of genes, which have already been shown to be related to melanoma development. Such as chemokines, which major role is to guide the migration of cells and mediate immune response are important for tumor invasion and metastatic behavior [56]. We also showed that CXCL5, CCL28 and chemokine-like protein FAM19A5 were significantly more expressed in MC compared to KC and FB (S2 Table). Additionally, a few genes, which regulate angiogenesis (semaphorins SEMA4C and SEMA6A, matrix metalloproteinases MMP8 and MMP17 [57]–[59] and are thereby essential for malignant processes, had a higher expression level in MC compared to KC and FB (S2 Table). ABCC2, ABCB5 and ABCB6, which were also highly expressed in MC compared to KC and FB (S2 Table), are the members of the ABC transporter family. ABC proteins have been shown to be involved in multidrug resistance in cancer treatment, but they also promote the pluripotency of embryonic cells and sustain the self-renewal of stem cells [60].

#### Melanocytes and inflammation

Susceptibility of cells to malignancies is strongly connected both inflammatory processes, but also their stem-cell-like properties. Inflammation influences cancer development at different levels - predisposing precancerosis, misdirect immune system, initiating invasion process etc. [61].

A glaring example about the relation of immune response and tumorogenesis is a family of tumor necrosis factors (TNFs) and their receptors. Being strongly engaged both in immune system modulation and apoptosis regulation, they trigger infiltration of inflammatory cells into tumorous tissue [62]. The cross-regulation of TNF and interferon regulatory factors have been proposed recently [63]. In line with this, the tumor necrosis factors receptors TNFRSF14, TNFRSF19 and interferon regulatory factors IFI6 and IRF4 were highly expressed in MC compared to KB and FB (S2 Table).

In our previous study [12] a quantitative real-time PCR (QRT-PCR) analysis based predesigned TaqMan Gene Expression Assays for selected interleukin 10 (IL10) family cytokine's genes showed the differential gene expression in cultivated MC relative to KC and FB. The data correlates well with the results obtained in the present study. For example IL20RA and IL20RB had significantly higher expression in KC compared to MC. Also IL22RA1 could be found in KC and not in MC, whereas IL22RA2 gene was expressed in whole skin but not in MC, KC or FB (S1 Table). Among the studied IL10 family cytokines (IL10, IL19, IL20, IL22, IL24, IL26, IL28B, IL29) and their receptors (IL10RA, IL10RB, IL20RA, IL20RB, IL22RA1, IL22RA2, IL28RA), IL24 was the most prominent cytokine in MC, which was hardly detectable in KC and FB (S2 Table). IL10 family of cytokines are responsible for host defense mechanisms and have both have both pro-inflammatory and anti-inflammatory roles [64].

Chronic inflammation is strongly connected to oxidative stress processes [65]. Melanin biosynthesis itself generates a large amount of free radicals [66], therefore it is crucial to have an efficient control system, which can balance the inflammatory process before it damages DNA or destroys the cell. For example, we found FOXO3 transcription factor, which coordinates reduction/oxidation balance in neural stem cells [67] and ATM, which assists cells in recognizing damaged, but is also modulating the antioxidant system, and glutathione peroxidase genes GPX3, GPX7 and GPX8, which regulate intracellular reactive oxygen species balance [68], to be upregulated in MC compared to KC and FB (S2 Table).

#### Stem cells-like properties

Cultured MC expressed a wide range of genes, characteristic for stem cells. Evidence shows that several pathways that are important in normal stem cells (BCL2 family genes, Notch, Sonic hedgehog and Wnt signaling pathways), may also act in cancer development [69], [70]. For instance, we saw the highest level of antiapoptotic BCL2 and BCL2A1 and stem cell factor inducer [71] RCAN1 expression level in MC when compared to KC, FB and the whole skin (S2 Table). Interestingly, CD200 was uniquely expressed in MC (Table 2). CD200 has been proposed to be a follicular stem cell marker, but its expression increases also with apoptosis and cancers overexpressing CD200 expand and metastasize more rapidly [72]. And, as mentioned above, the expression of S100 calcium-binding proteins, which are specifically expressed in cells with stem cell properties was increased in MC (S2 Table). Tumor cells and stem cells have similarities in their self-renewal process; they have extensive proliferative potential and stem cells are often a target for malignant genetic transformations [69]. These stem-cell-like properties have brought forth MC as a potential source for induced pluripotent stem cells (iPSCs) [73].

### Pathway analysis of melanocytes

**Figure 2:**
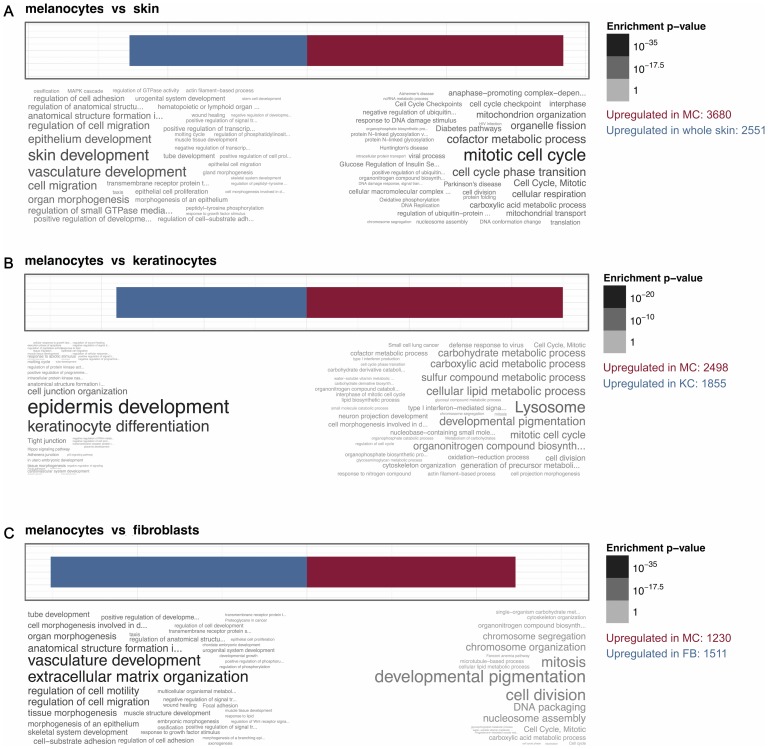
Comparative pathway analysis of MC and KC, FB and whole skin. Red plots indicate pathways, which were prominently expressed in MC. Blue plots mark pathways, which were downregulated in MC (A, B, C) and concomitantly upregulated in the whole skin (A), KC (B) and FB (C), respectively. The word sizes in wordclouds are defined by the p-values.

Pathway analysis of differentially expressed genes also described MC as active and intensively dividing cell population in cell culture (Fig. 2). We identified a number of pathways prevalent in MC, which were characteristic for ongoing regenerative process and could be related to cell dividing processes: genes regulating mitotic activity and cell cycle, DNA replication and packing, assembling and metabolism of different cellular components (cytoskeleton, structural macromolecules), formation of lysosome and Golgi complex etc. (Fig. 2). Consequently, gene expression profile corresponding to enhanced metabolic activity could be also observed.

Although the gene expression pattern analysis describes the cultured MC as highly proliferative cells we have to consider that the high proliferation rate is characteristic only to MC's cell in culture and does not reflect the actual situation in vivo. The cell culture conditions include less cell-cell contacts and a high level of growth factors reminiscent of active regenerative state (like in case of wound healing). Thus the analysis of gene expression pattern of cultured cells does not reflect the homeostatic state of the cells in a tissue but rather are expected to describe their response to the injury. On the other hand, a number of well-characterized cell type-specific genes could be readily identified from each cell population analyzed suggesting that the low-passage cultured cells have well retained their identity. Since the cell culture has remained as a gold standard in order to obtain sufficient amount of relatively homogenous cell populations for tissue engineering and toxicity testing, knowing the characteristic properties of cells in culture is instrumental for their further use in ex-vivo applications.

## Conclusions

In this study we have identified a number of genes and pathways, which are characteristic or unique for MC compared to KC and FB. We also demonstrated the difference between gene expression pattern of MC culture and the whole skin. The data presented provide an insight into the various possible roles of MC in the skin. As expected by the rapid growth in the cell culture, our differential gene expression and pathway analyses described MC as cells with a high proliferative capacity in vitro compared to KC an FB. That might suggest they have preserved the readiness to regenerate and some stem-cells-like properties more than KC and FB. However, these properties make MC the most vulnerable cells in the skin and provide an explanation to their increased susceptibility to harmful environment agents (eg UV exposure) and high incidence rate of malignant melanoma. On the other hand, the increased stem cell-like properties might give MC a good self-renewing potential and also advocate for their use as a potential source for induced pluripotent stem cells for therapeutic purposes.

## Supporting Information

S1 Fig **Multidimensional scaling blot for visualizing the level of similarity of individual samples in KC, MC, FB and whole skin (WS) groups.** The function plotMDS in edgeR package evaluates the similarity of MC, KC, FB and whole skin replicate samples that were used in our experiment. It calculates the root-mean-square of top 500 genes with largest absolute log2 fold change between the two samples, termed leading log2-fold-change. From the MDS plot it is quite clear that the distinction between the MC, KC and FB is prominent over the distinction of samples retrieved from the same individual.
(PDF)

S1 Table **RPKM values of genes we detected in MC, KC, FB and the whole skin.** A gene was considered as expressed only if the RPKM value in all samples of the corresponding cell type was above the cutoff (0.95). Conversely, the gene was labeled as not expressed if the RPKM value was below 0.95 in at least one of the samples.
(XLSX)

S2 Table **List of differentially expressed genes comparing MC to KC, FB and whole skin.**
(XLSX)

S3 Table **List of pathways (based on differentially expressed genes), prominent in MC compared to KC, FB and whole skin.**
(XLSX)
