# Correction: Melanocytes in the Skin – Comparative Whole Transcriptome Analysis of Main Skin Cell Types

**DOI:** 10.1371/journal.pone.0173792

**Published:** 2017-03-07

**Authors:** Paula Reemann, Ene Reimann, Sten Ilmjärv, Orm Porosaar, Helgi Silm, Viljar Jaks, Eero Vasar, Külli Kingo, Sulev Kõks

The following information is missing from the Data Availability section: Raw sequencing data are hosted at the Gene Expression Omnibus repository under the accession number GSE94655. This data can be found at the following URL: https://www.ncbi.nlm.nih.gov/geo/query/acc.cgi?acc=GSE94655.

## References

[1] Reemann P, Reimann E, Ilmjärv S, Porosaar O, Silm H, Jaks V, et al (2014) Melanocytes in the Skin—Comparative Whole Transcriptome Analysis of Main Skin Cell Types. PLoS ONE 9(12): e115717 doi: 10.1371/journal.pone.0115717 25545474 10.1371/journal.pone.0115717 PMC4278762

