# Supplementary figures and images for: Melanocytes in the Skin – Comparative Whole Transcriptome Analysis of Main Skin Cell Types
Source: PLoS One. 2014 Dec 29;9(12):e115717. doi: 10.1371/journal.pone.0115717 (PMC4278762; doi:10.1371/journal.pone.0115717)

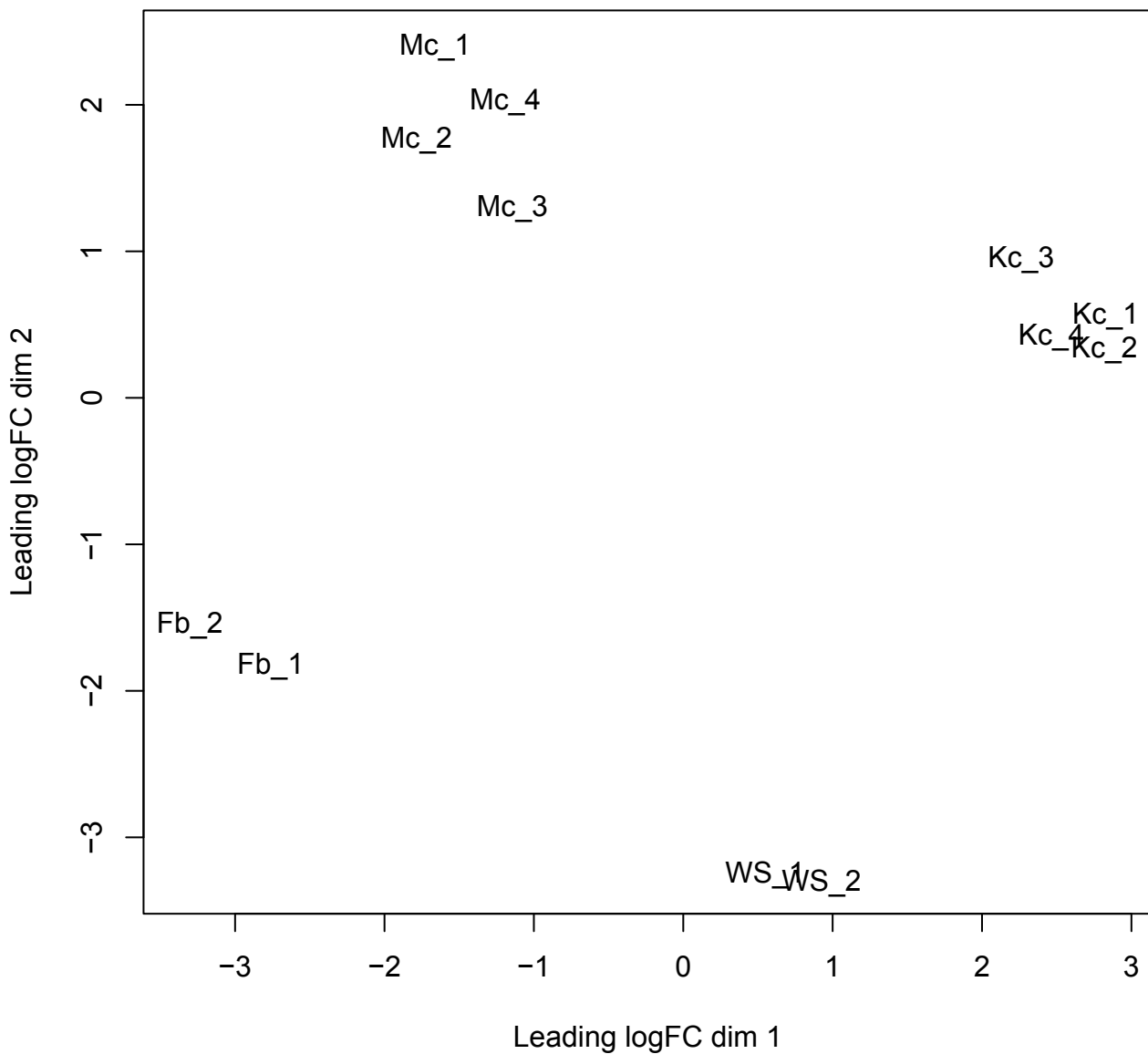

Supplement: S1 Fig — Multidimensional scaling blot for visualizing the level of similarity of individual samples in KC, MC, FB and whole skin (WS) groups. The function plotMDS in edgeR package evaluates the similarity of MC, KC, FB and whole skin replicate samples that were used in our experiment. It calculates the root-mean-square of top 500 genes with largest absolute log2 fold change between the two samples, termed leading log2-fold-change. From the MDS plot it is quite clear that the distinction between the MC, KC and FB is prominent over the distinction of samples retrieved from the same individual. (PDF) [file pone.0115717.s001.pdf]
